# Alcohol, tobacco and cannabis use are associated with job loss at follow-up: Findings from the CONSTANCES cohort

**DOI:** 10.1371/journal.pone.0222361

**Published:** 2019-09-09

**Authors:** Guillaume Airagnes, Cédric Lemogne, Pierre Meneton, Marie Plessz, Marcel Goldberg, Nicolas Hoertel, Yves Roquelaure, Frédéric Limosin, Marie Zins

**Affiliations:** 1 Hôpitaux Universitaires Paris Ouest, Department of Psychiatry and Addictology, AP-HP, Paris, France; 2 Faculté de Médecine, Université Paris Descartes, Sorbonne Paris Cité, Paris, France; 3 UMS 011, Population-based Epidemiological Cohorts, Inserm, Villejuif, France; 4 UMR 1168, VIMA, Inserm, Villejuif, France; 5 U 894, Centre Psychiatrie et Neurosciences, Inserm, Paris, France; 6 UMR 1142, Inserm, Sorbonne Université, Université Paris, Paris, France; 7 UMR 1085, Ester, Irest Inserm, Université d’Angers, Angers, France; Global Public Health, New York University, UNITED STATES

## Abstract

**Background:**

Substance use is more prevalent among unemployed subjects compared to employed ones. However, quantifying the risk subsequent of job loss at short-term according to substance use remains underexplored as well as examining if this association persist across various sociodemographic and occupational positions previously linked to job loss. We examined this issue prospectively for alcohol, tobacco, cannabis use and their combination, among a large population-based sample of men and women, while taking into account age, gender, overall health status and depressive symptoms.

**Methods:**

From the French population-based CONSTANCES cohort, 18,879 working participants were included between 2012 and 2016. At baseline, alcohol use disorder risk according to the Alcohol Use Disorders Identification Test (mild, dangerous, problematic or dependence), tobacco (non-smoker, former smoker, 1–9, 10–19, >19 cigarettes/day) and cannabis use (never, not in past year, less than once a month, once a month or more) were assessed. Employment status at one-year (working versus not working) was the dependent variable. Logistic regressions provided Odds Ratios(OR(95%CI)) of job loss at one-year, adjusting for age, gender, self-reported health and depressive state (measured with the Center of Epidemiologic Studies Depression scale). Stratified analyses were performed for education, occupational grade, household income, job stress (measured with the Effort-Reward Imbalance), type of job contract, type of work time and history of unemployment. In sensitivity analyses, employment status over a three-year follow-up was used as dependent variable.

**Results:**

Alcohol, tobacco and cannabis use were associated with job loss, from the second to the highest category: 1.46(95%CI:1.23–1.73) to 1.92(95%CI:1.34–2.75), 1.26(95%CI:1.09–1.46) to 1.78(95%CI:1.26–2.54) and 1.45(95%CI:1.27–1.66) to 2.68(95%CI:2.10–3.42), respectively, and with dose-dependent relationships (all p for trend <0.001). When introduced simultaneously, associations remained significant for the three substances without any between-substance interactions. Associations remained significant across almost all stratifications and over a three-year follow-up as well as after adjustment for all the sociodemographic and occupational factors.

**Conclusions:**

Alcohol, tobacco and cannabis use were independently associated with job loss at short-term, with dose-dependent relationships. This knowledge will help refining information and prevention strategies. Importantly, even moderate levels of alcohol, tobacco or cannabis use are associated with job loss at short-term and all sociodemographic and occupational positions are potentially concerned.

## Introduction

The costs associated with alcohol amount to more than 1% of the gross national product in high-income and middle-income countries, with the costs of social harm constituting a major proportion in addition to health costs [1]. The social costs of smoking are usually estimated to be even higher than those of alcohol use, and the social costs of illicit drugs could be around billions of Euros in European countries [2]. Substance use is among the leading preventable causes of premature death in the world and this burden is mainly driven by three substances: alcohol, tobacco and cannabis [3]. Regarding social harm, the association between substance use and unemployment, a major issue in most high-income countries, with a mean unemployment rate of 7.1% in Europe [4], is a critical question. Substance use is more prevalent among unemployed subjects compared to employed ones [5,6]. These findings are in accordance with an increased risk of substance use after job loss [7]. The onset of substance use after job loss might contribute to the increased morbidity and mortality observed among unemployed subjects [8]. However, substance use has been associated with detrimental consequences on occupational life and may predate job loss [5–10]. Several longitudinal studies have shown an association between substance use and the risk of subsequent job loss [11,12].

A large prospective study found an association between alcohol problematic use and subsequent unemployment during a 14-year follow-up [10]. An association between high alcohol consumption and job loss at 6 months was found among 658 at-risk drinkers [13]. High alcohol consumption and smoking were associated with long-term unemployment over a 4-year follow-up among 586 male blue-collar workers [14]. Heavy smoking (i.e. 20 cigarettes per day or more) was associated with an increased risk of being unemployed 4 years later, in men but not in women, among 5,355 employees [15]. A chronic use of cannabis in early adolescence was associated with a higher likelihood of unemployment at midlife among 548 participants [16]. Consistently, associations between cannabis use at age 18 and unemployment at age 40 were found among a large cohort of men [17]. Despite these evidence linking substance use and subsequent job loss, quantifying this risk of job loss at short-term in a large national population-based sample remains underexplored as well as examining if this association persist across various sociodemographic and occupational positions. This knowledge is critical to inform decision-makers in public health and occupational health and refine prevention measures by targeting individuals at higher risk for job loss and by using these findings as a potential motivational tool. However, to the best of our knowledge, no study has examined prospectively the risk of job loss at short-term according to substance use while stratifying by sociodemographic and occupational factors known to be related to substance use or job loss. These factors include sociodemographic variables such as age, gender, education level, marital status and household income, health-related factors such overall health status and depressive symptoms, and occupational factors such as occupational grade, job stress, type of job contract, type of work time and a past history of unemployment [5,6,18–27]. In addition, alcohol, tobacco and cannabis have rarely been studied together, which makes it difficult to know the specific role of each substance.

We took advantage of the CONSTANCES cohort to examine the prospective associations of alcohol, tobacco, cannabis use, and their combination, with job loss among a population-based sample of men and women large enough to allow stratifying for several sociodemographic and occupational factors. First, we searched for an increased risk of job loss according to substance use in the short term (i.e. at one year), and then whether this risk persists over a longer period of time (i.e. a three-year period). Second, we examined whether those associations persist within different subgroups defined based on sociodemographic and occupational factors previously linked to job loss, in order to identify those at greater risk for job loss associated with substance use. Because gender, age, overall health status and depressive symptoms may be potential confounders [5,15,25], we adjusted all the analyses for these variables. We hypothesized that the three substances will be associated with an increased risk of job loss at one-year and that these associations will hold across the different subgroups.

## Methods

### Participants

The CONSTANCES cohort includes volunteers aged 18–69 years at baseline according to a random sampling scheme stratified on age, gender, socioeconomic status and region of France [28]. Participants fulfill at baseline, and then annually, questionnaires on social and demographic characteristics, health-related behaviors and occupational conditions. All the questionnaires are available at www.constances.fr in both the original language and English. From the participants included between February 2012 and September 2016 (n = 81,997), the population study was restricted by the following inclusion criteria consistent with our objectives: being employed at baseline; having been included at least one year ago to allow sufficient follow-up duration; not being retired at one year. Thus, 18,879 participants have been included in the present study (**S1 Fig**). Depending on their inclusion date, the included participants had either a one-year follow-up (n = 10,145), a two-year follow-up (n = 6,454) or a three-year follow-up (n = 2,280). Precisely, among the participants who had only one year of follow-up, 84% (n = 8,556) were included too recently to have two years of follow-up. Similarly, among the participants who had two years of follow-up, 89% (n = 5,741) were included too recently to have three years of follow-up.

### Employment status

Employment status was self-reported at baseline and annually by answering the following question: “What is your current employment situation? 1) employed, including on sick leave, leave without pay or availability, maternity/paternity/adoption/parental leave; 2) job seeker or looking for a job; 3) retired or withdrawn from business; 4) in training (high school student, student, trainee, apprentice …; 5) does not work for health reasons (disability, chronic illness, etc.); 6) without employment; 7) other situation”. Participants employed were those who gave the response'1' or ‘4’. Retired participants were those who gave the response '3'. Participants experiencing job loss were those who reported at follow-up responses '2', '5', '6'or'7'.

### Substances

#### Alcohol use

We used the French version of the Alcohol Use Disorders Identification Test (AUDIT) to assess the risk of alcohol use disorder at baseline [29]. From the total score, we computed a categorical variable based on recommended AUDIT risk levels, as follows: 1) Mild (0–7), 2) Dangerous (8–15), 3) Problematic (16–19), and 4) Dependence (20–40). The two last categories were merged to ensure sufficient subsample size. The AUDIT has been also used after aggregating “Dangerous”, “Problematic” and “Dependence” categories and as binary variable as follows: mild risk (0–7) *versus* at-risk (8–40) alcohol use.

#### Tobacco use

Smoking status (i.e. never smoker, former smoker or current smoker) was self-reported at baseline. Among current smokers, daily tobacco consumption was collected in cigarettes per day. From these two variables, we computed a categorical variable to define: 1) Never smokers, 2) Former smokers, 3) Current light smokers (1–9 cigarettes per day),4) Current moderate smokers (10–19 cigarettes per day), and 5) Current heavy smokers (>19 cigarettes per day) [30]. Tobacco use has been also used after aggregating “current moderate smokers” and “current heavy smokers” and as binary variable by aggregating the first two categories (i.e. non-smokers) and the last three categories (i.e. current smokers).

#### Cannabis use

From three questions asked at baseline to characterize the frequency of cannabis consumption, we computed a categorical variable expressing the frequency of lifetime cannabis consumption as follows: 1) Never used; 2) No consumption during the previous 12 months; 3) Less than once a month; and 4) Once a month or more. Cannabis use has been also used as binary variable by aggregating the first two categories (i.e. no consumption during the year) and the last two categories (i.e. at least one consumption in the year).

### Adjustment variables

From the baseline questionnaires, we used age, gender, poor self-reported health and depressive state as covariables of adjustment. Age in years was used as a categorical variable in three modalities as follows: 1) <30; 2) ≥30 and <50 and 3) ≥50. Self-rated health status was measured from the following question: « How do you judge your general health compared to a person of your entourage of the same age? » to provide a proxy of overall health condition [31]. We computed a binary variable to define poor self-reported health (5 to 8, roughly corresponding to the 90^th^ percentile) versus good (1 to 4). Depressive symptoms were assessed with the Center of Epidemiologic Studies Depressive state scale (CESD) taking a global score ≥19 to signal clinically significant depressive state [32].

### Stratification variables

To search whether the association between substance use and subsequent job loss persist in different subgroups based on sociodemographic and occupational factors previously linked to job loss, we performed stratified analyses according to these factors. Thus, we examined whether these variables could act as effect modifiers. The following sociodemographic and occupational variables were used from the baseline questionnaires: age, gender, household income (i.e. <2800 *versus* ≥2800 euros per month), type of job contract (i.e. open-ended contract *versus* other type of contract), type of work time (i.e. full-time *versus* part-time), and education level based on the International Standard Classification of Education, as follows: levels 0 to 4 (up to upper secondary education or post-secondary non-tertiary education), levels 5 and 6 (short-cycle tertiary education and Bachelor’s or equivalent level), and levels 7 and 8 (Master’s or equivalent level and Doctoral or equivalent level) [33]. Occupational grade was used in three categories as follows: 1) Blue-collar worker, craftsman or employee; 2) Intermediate worker; 3) Executive. Regarding participants still in initial training (n = 379; i.e. 2% of the entire sample), we used the highest occupational grade of their parents, whether it was their father or their mother. As a standardized measure of job stress, the effort-reward imbalance was assessed at baseline [26]. We used a binary variable based on tertiles as follows: 1) first and second tertiles (i.e. low job stress), and 2) third tertile (i.e. high job stress). Regarding history of unemployment, we used data from national administrative databases that are annually linked with the CONSTANCES cohort [28]. From these data, we computed a binary variable for past time unemployed in the three years before inclusion.

### Statistical analysis

We first used binomial logistic regressions to compute risks of subsequent job loss according to substance use at baseline. Mild alcohol use risk, non-smoking and never used were chosen as reference for alcohol, tobacco and cannabis use, respectively. All the models were adjusted for age, gender, self-reported health and depressive symptoms. Results are presented as estimated Odds Ratios (OR) with their 95% Confidence Intervals (CI).

Regarding our aim to search for increased risk of job loss according to alcohol, tobacco and cannabis use and their combination over a one-year follow-up, we first introduced alcohol, tobacco and cannabis use separately in the models. Dose-dependent relationships were searched by introducing the substances as continuous variables. Interactions between each substance and each covariable were examined. Then, to further explore the specific and common effects of these three substances, we introduced simultaneously alcohol, tobacco and cannabis use in the same model. We primarily chose to keep at least three categories of use for all substances to inform on a potential gradient in their relations with job loss. However, in order to search for statistical significance while preventing from lack of power due to small sample sizes in some categories, this analysis has also been made after aggregating the “dangerous” and “problematic or dependence” categories regarding alcohol use, and the “moderate” and “heavy” categories regarding tobacco use. Finally, interactions between the three substances were examined by testing in pairs all the interactions in three different models. In this analysis, alcohol, tobacco and cannabis use were introduced in the models as binary variables in order to ease the search and interpretation of potential between-substance interactions.

Regarding our aim to search for at-risk subgroups, separate stratified analyses for each stratification variable were computed. Alcohol, tobacco and cannabis use were introduced in the models as binary variables, in order to ease the search and interpretation of potential effect modifiers.

Sensitivity analyses were conducted. First, since some subjects could have be aware of their risk of job loss one year before, we planned to search for similar associations over a longer duration of follow-up in order to take into account for this potential bias of reverse causality. Thus, we performed generalized estimating equations to search for similar associations while taking into account data at one-year, two-year and three-year follow-up. Alcohol, tobacco and cannabis use were entered successively in the models with occupational status as outcome. Interactions between duration of follow-up and each substance were examined. Second, we adjusted our analyses for all the covariables (i.e. age, gender, depressive symptoms, self-reported health, education level, occupational grade, household income, job stress, type of contract, type of work time, past time unemployed). Third, we examined whether the associations between substance use and subsequent risk of job loss could persist while excluding unemployed participants at follow-up with another status than job seeking (i.e. those who do not work for health reasons (n = 103), those without employment but not in job seeking (n = 54) and those in other situations (n = 383)).

Included subjects had complete data regarding the variables of interest (**S1 Fig**). We had missing data for the other variables (from 0.5% to 10.3%), except for age, gender and occupational grade. Assuming a missing at random mechanism, we used stochastic regression imputations rather than complete-case analysis to limit the risk of selection bias [34].

Statistical significance was determined using a two-sided alpha a priori set at 0.05 and analyses were performed with IBM Statistics for Windows, Version 22.0, Released 2013 (Armonk, NY: IBM Corp).

## Results

### Participants’ characteristics

Among the 18,879 participants, 1,129 (6.0%) experienced job loss at one-year. Their characteristics are displayed in **Table 1**. As expected, job loss was more prevalent among women, participants with low education, low occupational grade, low income, depression, poor self-reported health, job stress, other type of contract than open-ended, part-time job and past-time unemployed.

**Table 1:** Characteristics of the 18,879 included participants from the CONSTANCES cohort and according to their employment status at one-year follow-up.

	TOTAL		EMPLOYED AT ONE-YEAR		JOB LOSS AT ONE-YEAR	
N (%)	18,879 (100%)		17,750 (94.0%)		1,129 (6.0%)	
SUBSTANCE USE	N	%	N	%	N	%
**Alcohol use**^**a**^						
Mild	16344	86.6	15438	87.0	906	80.2
Dangerous	2235	11.8	2050	11.5	185	16.4
Problematic or dependence	300	1.6	262	1.5	38	3.4
**Tobacco use**^**b**^						
Non-smokers	8774	46.5	8337	47.0	437	38.7
Former smokers	6374	33.8	5995	33.8	379	33.6
Light smokers	2032	10.8	1871	10.5	161	14.3
Moderate smokers	1290	6.8	1177	6.6	113	10.0
Heavy smokers	409	2.2	370	2.1	39	3.5
**Cannabis use**						
Never used	9788	51.8	9327	52.5	461	40.8
No consumption during the previous 12 months	7513	39.8	7017	39.5	496	43.9
Less than once a month	784	4.2	711	4.0	73	6.5
Once a month or more	794	4.2	695	4.0	99	8.8
**SOCIODEMOGRAPHIC FACTORS**						
**Age** (years)						
Less than 30	2245	11.9	2012	11.3	233	20.6
Between 30 and 50	10620	56.3	10093	56.9	527	46.7
More than 50	6014	31.9	5645	31.8	369	32.7
**Gender**						
Men	8823	46.7	8340	47.0 48.0	483	42.8
Women	10056	53.3	9410	53.0	646	57.2
**Education ISCED classification**						
Levels 0 to 4	5875	31.1	5440	30.6	435	38.5
Levels 5 and 6	7523	39.8	7103	40.0	420	37.2
Levels 7 and 8	5481	29.0	5207	29.3	274	24.3
**Occupational grade**						
Blue-collar worker, craftsman or employee	5758	30.5	5254	29.6	504	44.6
Intermediate worker	5732	30.4	5458	30.7	274	24.3
Executive	7389	39.1	7038	39.7	351	31.1
**Household income** (euros per month)						
Less than 2800	5750	30.5	5201	29.3	549	48.6
More than 2800	13129	69.5	12549	70.7	580	51.4
**DEPRESSIVE STATE**^**c**^						
No	16421	87.0	15566	87.7	855	75.7
Yes	2458	13.0	2184	12.3	274	24.3
**SELF-REPORTED HEALTH** ^**d**^						
Good	17116	90.7	16163	91.1	953	84.4
Poor	1763	9.3	1587	8.9	176	15.6
**OCCUPATIONAL FACTORS**						
**Effort-reward imbalance ratio**^**e**^						
First and second tertiles	13093	69.4	12383	69.8	710	62.9
Third tertile	5786	30.6	5367	30.2	419	37.1
**Type of contract**						
Open-ended contract	15886	84.1	15123	85.2	763	67.6
Other type of contract	2993	15.9	2627	14.8	366	32.4
**Type of work time**						
Full-time	15940	84.4	15135	85.3	805	71.3
Part-time	2939	15.6	261	14.7	324	28.7
**Past time unemployed in the last three years**						
No	17221	91.2	16426	92.5	795	70.4
Yes	1658	8.8	1324	7.5	334	29.6

ISCED: 2011 International Standard Classification of Education

^a^ Categories are defined from Alcohol Use Disorders Identification scores as follows: Mild (0–7), Dangerous (8–15), Problematic (16–19) and Dependence (20–40)

^b^ Categories of current smokers are defined as follows: Light (1 to 9 cigarettes per day), Moderate (10 to 19) and Heavy (>19) consumers

^c^ A total score at the Center of Epidemiologic Studies Depression scale ≥19 defined a depressive state

^d^ Self-reported health was used as a binary variable from an 8-points Likert scale

^e^ Computed from 7 items regarding rewards and from 3 items regarding efforts and with all the items assessed on a 4-points Likert scale.

### Associations between alcohol, tobacco and cannabis use and job loss at one-year

**Fig 1:**
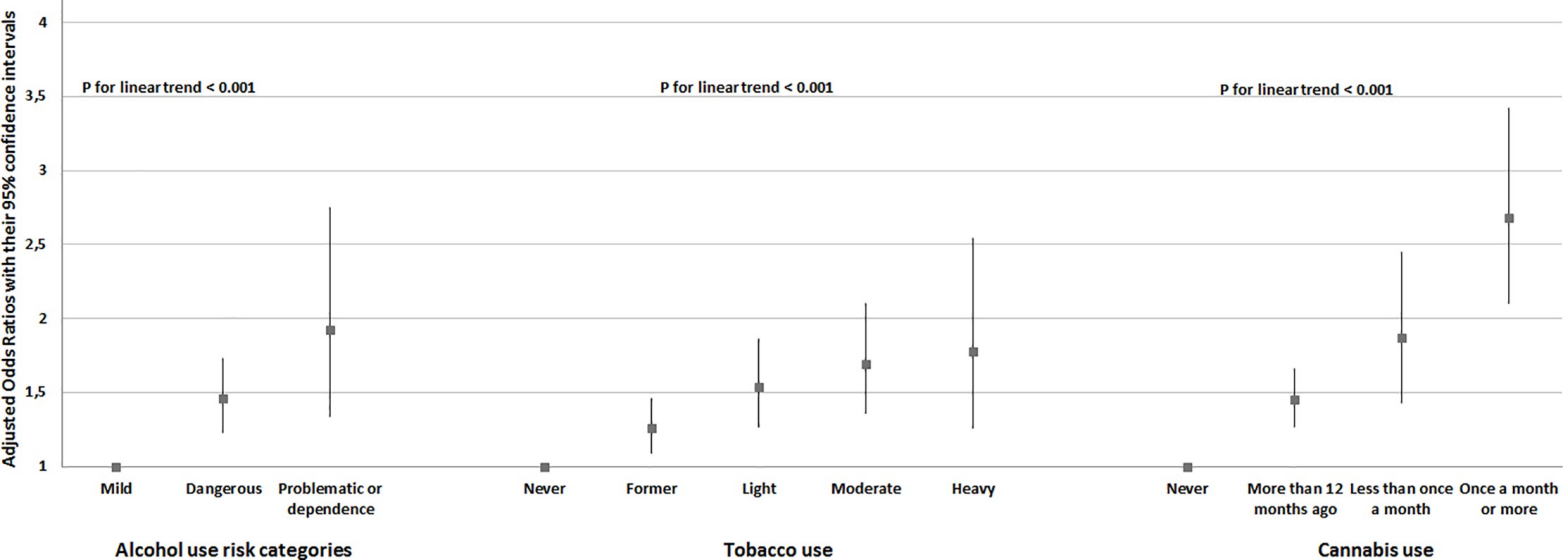
Risk of job loss at one-year according to substance use, adjusting for age, gender, self-reported health and depressive symptoms, among 18,879 participants from the CONSTANCES cohort.

When introduced separately, alcohol, tobacco and cannabis use were positively associated with job loss at one-year after adjusting for age, gender, depressive symptoms and self-rated health (**Table 2 and S1 Table**). We found dose-dependent relationships for alcohol (OR = 1.42(95%CI:1.25,1.62)), tobacco (OR = 1.19(95%CI:1.13,1.26)) and cannabis use (OR = 1.39(95%CI:1.29,1.50)) (**Fig 1**). We did not found any interaction between alcohol, tobacco and cannabis use and adjustment variables, excepting with depressive state for tobacco use and with age for cannabis use. More precisely, we found an interaction between depressive state and being a heavy smoker; OR = 0.33(95%CI = 0.14,0.76). In stratified analyses according to depressive state, all the associations were significant and in the same direction, except for depressed heavy smoker most probably due to too small sample size (only 8 subjects experienced job loss in this subgroup (n = 101), compared to at least 30 in the other subgroups) (**S2 Table**). We also found an interaction between age more than 50 and having used cannabis more than 12 months ago; OR = 0.63(95%CI:0.42,0.96) and an interaction between age from 30 to 50 and having used cannabis more than 12 months ago; OR = 0.58 (95%CI:0.39,0.86)). In stratified analyses according to age, all the associations were significant and in the same direction, except for consumers less than once month aged over 50, most probably due to too small sample size (only 8 subjects experienced job loss in this subgroup (n = 74), compared to at least 30 in the other subgroups) (**S3 Table**).

**Table 2:** Associations between alcohol, tobacco and cannabis use and job loss at one-year among 18,879 participants from the CONSTANCES cohort, adjusting for age, gender, self-reported health and depressive symptoms.

Type of model	Each substance successively entered				All substances simultaneously entered			
	OR	95%CI		p value	OR	95%CI		p value
**Alcohol use**^**a**^								
Dangerous	**1.46**	**1.23**	**1.73**	**<0.001**	**1.22**	**1.02**	**1.46**	**0.030**
Problematic or Dependence	**1.92**	**1.34**	**2.75**	**<0.001**	1.42	0.98	2.05	0.067
**Tobacco use**^**b**^								
Former smoker	**1.26**	**1.09**	**1.46**	**0.002**	1.08	0.93	1.26	0.320
Light smoker	**1.54**	**1.27**	**1.86**	**<0.001**	1.17	0.96	1.44	0.128
Moderate smoker	**1.69**	**1.36**	**2.10**	**<0.001**	**1.29**	**1.02**	**1.63**	**0.034**
Heavy smoker	**1.78**	**1.26**	**2.54**	**0.001**	1.33	0.92	1.92	0.126
**Cannabis use**^**c**^								
Consumption more than 12 months ago	**1.45**	**1.27**	**1.66**	**<0.001**	**1.35**	**1.17**	**1.56**	**<0.001**
Less than once a month	**1.87**	**1.43**	**2.45**	**<0.001**	**1.62**	**1.22**	**2.15**	**0.001**
Once a month or more	**2.68**	**2.10**	**3.42**	**<0.001**	**2.19**	**1.68**	**2.87**	**<0.001**

OR: Odds ratios; 95%CI: Confidence interval at 95%

^a^ Categories are defined from Alcohol Use Disorders Identification scores as follows: Mild (0–7), Dangerous (8–15), Problematic (16–19) and Dependence (20–40), with Mild category as reference

^b^ Categories of current smokers are defined as follows: Light (1 to 9 cigarettes per day), Moderate (10 to 19) and Heavy (>19) consumers, with never smokers as reference category

^c^ Reference category is never use. Adjustments variables were as follows: gender, age in three categories (<30; ≥30 and <50 and ≥50), self-reported health was used as a binary variable from an 8-points Likert scale, and depressive state defined as a total score ≥19 at the Center for Epidemiologic Studies Depression (CESD). Significant associations are presented in bold (i.e. p<0.05).

When introduced simultaneously, alcohol, tobacco and cannabis use were still positively associated with job loss for alcohol consumers having a dangerous use, for moderate smokers and for all the categories of cannabis consumers after adjustment for age, gender, depressive symptoms and self-reported health (**Table 2 and S1 Table).** We found dose-dependent relationships for alcohol (OR = 1.20(95%CI:1.05,1.38)), tobacco (OR1.08 = (95%CI:1.02,1.15)) and cannabis use (OR = 1.30(95%CI:1.20,1.41)). While performing this analysis after aggregating the two last categories of alcohol and tobacco use, we found significant associations between these categories and job loss and still with dose-dependent relationships for tobacco use for which there were more than 2 categories left (**S4 Table**).

We found no interactions between pairs of substances: OR = 0.74 (95%CI:0.51,1.07) with p = 0.113 for the interaction between alcohol and cannabis use, OR = 1.09(95%CI:0.79,1.51) with p = 0.589 for the interaction between alcohol and tobacco use, and OR = 1.09(95%CI:0.75,1.58) with p = 0.664 for the interaction between cannabis and tobacco use. Odds ratios of job loss while using substance use as binary variables are presented in **S5 Table**.

### Stratifications for sociodemographic and occupational factors

In stratified analyses for sociodemographic and occupational factors, all the associations between substance use and job loss were positive (**Table 3**). Over a total of 27 stratifications, significant associations persisted in all strata among 20 of them. Regarding alcohol use, the only sub-groups in which the associations were not significant were the youngest (OR = 1.30(95%CI:0.95,1,79)), those with low income (OR = 1.18(0.94,1.49)), those at part-time (OR = 1.34(95%CI:0.94,1.91)) and those with history of unemployment (OR = 1.00(95%CI:0.73,1.38)). These associations were significant in all these sub-groups for the two other substances (i.e. tobacco and cannabis). Regarding tobacco use, the only sub-groups in which the associations were not significant were the oldest (OR = 1.19(95%CI:0.90,1.57)), those with the highest occupational grade (OR = 1.20(95%CI:0.92,1.57)) and those with the highest income (OR = 1.23(95%CI:0.99,1.52)). These associations were significant in all these sub-groups for the two other substances (i.e. alcohol and cannabis). Regarding cannabis use, associations were significant in all strata for all the stratifications.

**Table 3:** Associations between alcohol, tobacco and cannabis use and job loss at one-year among 18,879 participants from the CONSTANCES cohort, adjusting for age, gender, self-reported health and depressive symptoms, and while stratifying for sociodemographic and occupational factors.

	Type of substance use											
	Alcohol^a^				Tobacco^b^				Cannabis^c^			
SOCIODEMOGRAPHIC FACTORS	OR	95%CI		p value	OR	95%CI		p value	OR	95%CI		p value
**Age** (in years)												
Less than 30	1.30	0.95	1.79	0.105	**1.86**	**1.40**	**2.47**	**<0.001**	**1.51**	**1.11**	**2.04**	**0.008**
Between 30 and 50	**1.48**	**1.17**	**1.89**	**0.001**	**1.47**	**1.21**	**1.79**	**<0.001**	**1.99**	**1.55**	**2.56**	**<0.001**
More than 50	**1.82**	**1.36**	**2.44**	**<0.001**	1.19	0.90	1.57	0.224	**2.84**	**1.73**	**4.66**	**<0.001**
**Gender**												
Men	**1.55**	**1.26**	**1.91**	**<0.001**	**1.71**	**1.39**	**2.10**	**<0.001**	**1.96**	**1.52**	**2.53**	**<0.001**
Women	**1.45**	**1.12**	**1.87**	**0.005**	**1.30**	**1.08**	**1.57**	**0.006**	**1.78**	**1.37**	**2.31**	**<0.001**
**Education level** (2011 ISCED classification)												
Levels 0 to 4	**1.45**	**1.12**	**1.87**	**0.004**	**1.51**	**1.22**	**1.88**	**<0.001**	**1.82**	**1.33**	**2.50**	**<0.001**
Levels 5 and 6	**1.44**	**1.09**	**1.91**	**0.011**	**1.33**	**1.06**	**1.68**	**0.015**	**1.94**	**1.44**	**2.62**	**<0.001**
Levels 7 and 8	**1.64**	**1.20**	**2.24**	**0.002**	**1.41**	**1.05**	**1.90**	**0.023**	**1.79**	**1.27**	**2.53**	**0.001**
**Occupational grade**												
Blue-collar worker, craftsman or employee	**1.37**	**1.07**	**1.76**	**0.013**	**1.52**	**1.25**	**1.86**	**<0.001**	**1.78**	**1.35**	**2.36**	**<0.001**
Intermediate worker	**1.43**	**1.02**	**2.00**	**0.040**	**1.45**	**1.09**	**1.92**	**0.010**	**1.50**	**1.01**	**2.24**	**0.045**
Executive	**1.76**	**1.34**	**2.31**	**<0.001**	1.20	0.92	1.57	0.187	**2.30**	**1.69**	**3.13**	**<0.001**
**Household income**(in euros per month)												
Less than 2800	1.18	0.94	1.49	0.164	**1.54**	**1.27**	**1.85**	**<0.001**	**1.63**	**1.28**	**2.07**	**<0.001**
More than 2800	**1.82**	**1.46**	**2.27**	**<0.001**	1.23	0.99	1.52	0.054	**1.92**	**1.45**	**2.55**	**<0.001**
**OCCUPATIONAL FACTORS**	**OR**	**95%CI**		**p value**	**OR**	**95%CI**		**p value**	**OR**	**95%CI**		**p value**
**Effort-reward imbalance ratio**^**d**^												
First and second tertiles	**1.45**	**1.18**	**1.78**	**<0.001**	**1.45**	**1.21**	**1.72**	**<0.001**	**1.85**	**1.48**	**2.32**	**<0.001**
Third tertile	**1.61**	**1.24**	**2.09**	**<0.001**	**1.50**	**1.20**	**1.88**	**<0.001**	**1.87**	**1.37**	**2.55**	**<0.001**
**Type of contract**												
Open-ended contract	**1.50**	**1.12**	**1.82**	**<0.001**	**1.43**	**1.20**	**1.69**	**<0.001**	**1.77**	**1.40**	**2.25**	**<0.001**
Other type of contract	**1.47**	**1.10**	**1.96**	**0.010**	**1.51**	**1.19**	**1.93**	**0.001**	**1.57**	**1.18**	**2.10**	**0.002**
**Type of work time**												
Full-time	**1.56**	**1.30**	**1.87**	**<0.001**	**1.47**	**1.25**	**1.72**	**<0.001**	**1.82**	**1.47**	**2.24**	**<0.001**
Part-time	1.34	0.94	1.91	0.105	**1.50**	**1.16**	**2.01**	**0.002**	**1.71**	**1.18**	**2.49**	**0.005**
**History of unemployment during the last three years**												
No	**1.63**	**1.35**	**1.97**	**<0.001**	**1.34**	**1.13**	**1.59**	**0.001**	**1.73**	**1.38**	**2.17**	**<0.001**
Yes	1.00	0.73	1.38	0.990	**1.37**	**1.05**	**1.79**	**0.019**	**1.59**	**1.15**	**2.20**	**0.006**

OR: Odd ratio; 95%CI: Confidence interval at 95%; ISCED: 2011 International Standard Classification of Education

^a^ Categories are defined from Alcohol Use Disorders Identification scores as follows: Mild (0–7), Dangerous, Problematic and Dependence (8–40), with Mild category as reference

^b^ Non-smokers is defined as reference category compared to former and current smokers

^c^ Never used is defined as reference category compared to having already used

^d^ Computed from 7 items regarding rewards and from 3 items regarding efforts and with all the items assessed on a 4-points likert scale. Adjustments variables were as follows: gender, age in three categories (<30; ≥30 and <50 and ≥50), self-reported health was used as a binary variable from an 8-points Likert scale, and depressive state defined as a total score ≥19 at the Center for Epidemiologic Studies Depression (CESD). Significant associations are presented in bold (i.e. p<0.05).

### Sensitivity analysis

In generalized estimating equations over a three-year duration of follow-up, all the associations between substance use and job loss remained positive and with similar effects sizes (**S6 Table**). We found no time by substance interaction for alcohol (OR = 0.98(95%CI:0.80,1.19); p = 0.827), nor for tobacco (OR = 0.99(95%CI:0.83,1.18); p = 0.886) and nor for cannabis use (OR = 0.86(95%CI:0.69,1.08); p = 0.185).

Associations between alcohol, tobacco and cannabis use and subsequent job loss persist after adjustment for all the covariables (**S7 Table**).

Finally, we excluded successively, and then simultaneously, the three types of unemployed participants at follow-up having another status than job seeking. All these analyses provided similar results.

## Discussion

### Summary of the results

Our main objective was to examine the prospective associations between alcohol, tobacco, cannabis use and their combination with job loss at one-year, among a large population-based sample of men and women, while adjusting for age, gender, self-reported health and depressive symptoms. We found that alcohol, tobacco and cannabis use were independently associated with job loss at one-year, showing dose-dependent relationships. We further sought to examine those associations while stratifying for a broad range of sociodemographic and occupational factors previously linked to job loss. Since the associations remained significant across almost all stratifications, all sociodemographic and occupational positions are potentially concerned. Finally, associations remained positive and with similar effect sizes over a three-year follow-up.

### Strengths and limitations

To our knowledge, this is the first study to examine the prospective associations of each substance use and their combination and subsequent job loss in the short term in a large national population-based cohort. Over the inclusion period (i.e. 2012 to 2016), the unemployment rate at baseline among the 81,997 participants from the CONSTANCES cohort (11%, including 6.1% who are actively seeking employment) is close to the mean unemployment rate in France (10.2%) while taking the definition of the international labour office [35]. In addition, sociodemographic and occupational factors associated with job loss in CONSTANCES were similar to those described in the general population [5,6,15,16,25–27,36]. Thanks to the large sample, analyses were stratified for several individual and occupational factors to examine whether these associations were specific to subgroups or rather a general phenomenon. In particular, job stress was assessed with a well-validated standardized tool. Finally, all our analyses were adjusted for potential confounders including age, gender, self-reported health and depressive symptoms.

Our study has several limitations. Firstly, even if the CONSTANCES cohort randomly recruited its participants, this population is not representative of the general population due to selection effects associated with voluntary participation. Thus, our findings may not apply to the same extent to other settings. However, the associations were consistent in most stratified analyses, suggesting that potential selection biases related to these sociodemographic and occupational factors may be unlikely to substantially affect our conclusions. Secondly, the listwise deletion of individuals who had missing data for the main variables of interest led to a decrease in statistical power and, potentially, the selection of subjects less likely to display severe substance use or to loss their job. In addition, depression and overall health status might act not only as confounders but also as mediators. Therefore our results might have underestimated the strength of the associations. This issue might explain the lack of significance for some categories of the exposure with small sample. For instance, while introducing simultaneously in the same model the three substances, problematic alcohol use or dependence, as well as heavy smoking, were no longer associated with the risk of job loss. Noteworthy, dose-dependent relationships were found for the three substances in all the models and there were again significant associations after aggregating these categories with the lower category of use. Furthermore, compared with models including only one substance, simultaneously including the three substances did not affect much the association of cannabis with job loss (mean decrease of the odds ratios of 13%). In contrast, the decreases of odds ratios for alcohol and tobacco use were higher but of similar magnitude. Specifically, the decreases were of 21% and of 22% for alcohol and tobacco use, respectively. This suggests that the association of tobacco with job loss might be partially confounded by the use of the two other substances, but not to a greater extent than alcohol. Thirdly, the prospective design did not rule out the possibility of reverse causality. However, although some participants who experienced job loss at one-year might have anticipated this condition, similar results were found in analyses over a three-year follow-up.

### Explanatory hypotheses

Several explanatory hypotheses can be proposed concerning the links between substance use and subsequent job loss. Regarding alcohol use, acute effects rarely go unnoticed, such as disinhibition following intoxication, or tremors, sweats and irritability following withdrawal. In addition, chronic alcohol use may induce long-term neuropsychological impairment such as memory, concentration and attention disorders. All these symptoms may decrease the ability to perform work-related tasks. Regarding tobacco use, smoking is associated with psychological distress including anxiety and irritability which could contribute to a decreased productivity [37]. Moreover, smoking is time-consuming. Regarding the association between former smoking and job loss, they might be explained, at least partially, by the potential increased vulnerability of former smokers to environmental stressors. Anxiety, which is associated with loss productivity [38], has been found to be more prevalent in former smokers than in non-smokers, even after long periods of cessation [39]. Regarding cannabis, its use is associated with symptoms that could substantially interfere with work tasks, such as attentional disorders and persecution delusions [40]. Physical symptoms are also particularly noticeable for the entourage such as the hyperemesis syndrome. Chronic use may also lead to amotivational syndrome and impaired decision-making [41].

### Public health implications and future research

As regards information, important messages can be delivered to the population; for instance even moderate levels of alcohol, tobacco or cannabis use are associated with job loss and all sociodemographic and occupational positions are potentially concerned. As regards prevention, measures could be taken within the companies to limit the risks of substance use, for example by no longer offering alcohol in the canteen, and by extending the smoking ban to the whole company (e.g. outdoor work). Preventive and occupational medicine could also play a substantial role by screening at-risk subjects to prevent substance use and its detrimental consequences on occupational life. Moreover, informing on these potential consequences might be a promising motivational tool. Importantly, being excluded from employment is associated with a significant deterioration in physical and psychological health, and substance use makes it more difficult to return to employment. Since unemployment concern a large panel of the general population, from a public health perspective it is of paramount interest to prevent entry into this vicious circle. Future studies should examine which types of jobs might be more concerned than others. Finally, future studies should test whether interventions aiming at preventing or decreasing substance use improve job retention.

## Supporting information

S1 Fig Flow chart. (DOCX)

S1 Table Associations between alcohol, tobacco and cannabis use and job loss at one-year among 18,879 participants from the CONSTANCES cohort, adjusting for age, gender, self-reported health and depressive symptoms. (DOCX)

S2 Table Associations between tobacco use and job loss at one-year among 18,879 participants from the CONSTANCES cohort, adjusting for age, gender and self-reported health while stratifying for depressive state. (DOCX)

S3 Table Associations between cannabis use and job loss at one-year among 18,879 participants from the CONSTANCES cohort, adjusting for gender, depressive state and self-reported health while stratifying for age. (DOCX)

S4 Table Associations between alcohol, tobacco and cannabis use and job loss at one-year among 18,879 participants from the CONSTANCES cohort, adjusting for age, gender, self-reported health and depressive symptoms, with all substances simultaneously entered and while aggregating “dangerous” and “problematic or dependence” categories regarding alcohol use, as well as the “moderate” and “heavy” categories regarding tobacco use. (DOCX)

S5 Table Associations between alcohol, tobacco and cannabis use and job loss at one-year among 18,879 participants from the CONSTANCES cohort, adjusting for age, gender, self-reported health and depressive symptoms while using substances as binary variables. (DOCX)

S6 Table Associations between alcohol, tobacco and cannabis use and job loss over a three-year follow-up among 18,879 participants from the CONSTANCES cohort, adjusting for age, gender, poor self-reported health and depressive state. (DOCX)

S7 Table Associations between alcohol, tobacco and cannabis use and job loss at one-year among 18,225 participants from the CONSTANCES cohort, adjusting for age, gender, education level, occupational grade, household income, effort-reward imbalance ratio, type of contract, type of work time, past time unemployed in the past three years, depressive symptoms and self-reported health. (DOCX)
