# Supplementary material for: Alcohol, tobacco and cannabis use are associated with job loss at follow-up: Findings from the CONSTANCES cohort
Source: PLoS One. 2019 Sep 9;14(9):e0222361. doi: 10.1371/journal.pone.0222361 (PMC6733456; doi:10.1371/journal.pone.0222361)
Supplement: S1 Fig — (DOCX) [file pone.0222361.s001.docx]

**Missing data regarding employment status at one-year follow-up**

**n=8,312**

**Missing data regarding employment status at baseline**

**n=3,793**

**Missing data regarding variables of interest**

**n=4,843**

Alcohol use: n=3,538
Tobacco use: n=952
Cannabis use: n=967

**Not at work at baseline**

n=29,603

Job seeking: n=4,808

Retired: n=17,215

Others situations: n= 3,787

**Included too recently in the cohort to have a one-year follow-up**

n=19,512

**Participants at work at baseline**n=52,394

**Participants included in the CONSTANCES cohort between February 2012 and September 2016**

n=81,997

**S1 Fig. Flow chart**

**Included participants**

n=18,879

**Participants at work at baseline
with a follow-up at one-year without a transition to retirement**

n=23,722

**Transition to retirement at one year**

n=848

**Participants at work at baseline
with a follow-up at one-year**

n=24,570
