# Supplementary material for: Alcohol, tobacco and cannabis use are associated with job loss at follow-up: Findings from the CONSTANCES cohort
Source: PLoS One. 2019 Sep 9;14(9):e0222361. doi: 10.1371/journal.pone.0222361 (PMC6733456; doi:10.1371/journal.pone.0222361)
Supplement: S1 Table — (DOCX) [file pone.0222361.s002.docx]

| **Type of model** | **Each substance successively entered** | | | | | | | | | **All substances simultaneously entered** | | |
| --- | --- | --- | --- | --- | --- | --- | --- | --- | --- | --- | --- | --- |
|  | **Alcohol** | | | **Tobacco** | | | **Cannabis** | | |  |  |  |
|  | **OR** | **95%CI** | | **OR** | **95%CI** | | **OR** | **95%CI** | | **OR** | **95%CI** | |
| **SUBSTANCE USE** |  |  |  |  |  |  |  |  |  |  |  |  |
| **Alcohol use^a^** |  |  |  |  |  |  |  |  |  |  |  |  |
| Dangerous | **1.46** | **1.23** | **1.73** |  |  |  |  |  |  | **1.22** | **1.02** | **1.46** |
| Problematic or Dependence | **1.92** | **1.34** | **2.75** |  |  |  |  |  |  | 1.42 | 0.98 | 2.05 |
|  |  |  |  |  |  |  |  |  |  |  |  |  |
| **Tobacco use^b^** |  |  |  |  |  |  |  |  |  |  |  |  |
| Former smoker |  |  |  | **1.26** | **1.09** | **1.46** |  |  |  | 1.08 | 0.93 | 1.26 |
| Light smoker |  |  |  | **1.54** | **1.27** | **1.86** |  |  |  | 1.17 | 0.96 | 1.44 |
| Moderate smoker |  |  |  | **1.69** | **1.36** | **2.10** |  |  |  | **1.29** | **1.02** | **1.63** |
| Heavy smoker |  |  |  | **1.78** | **1.26** | **2.54** |  |  |  | 1.33 | 0.92 | 1.92 |
|  |  |  |  |  |  |  |  |  |  |  |  |  |
| **Cannabis use^c^** |  |  |  |  |  |  |  |  |  |  |  |  |
| Consumption more than 12 months ago |  |  |  |  |  |  | **1.45** | **1.27** | **1.66** | **1.35** | **1.17** | **1.56** |
| Less than once a month |  |  |  |  |  |  | **1.87** | **1.43** | **2.45** | **1.62** | **1.22** | **2.15** |
| Once a month or more |  |  |  |  |  |  | **2.68** | **1.10** | **3.42** | **2.19** | **1.68** | **2.87** |
|  |  |  |  |  |  |  |  |  |  |  |  |  |
| **SOCIODEMOGRAPHIC FACTORS** |  |  |  |  |  |  |  |  |  |  |  |  |
| **Age** (in years; reference category: Less than 30) |  |  |  |  |  |  |  |  |  |  |  |  |
| Between 30 and 50 | **0.48** | **0.41** | **0.56** | **0.45** | **0.38** | **0.53** | **0.51** | **0.43** | **0.60** | **0.51** | **0.43** | **0.61** |
| More than 50 | **0.60** | **0.51** | **0.72** | **0.57** | **0.48** | **0.68** | **0.71** | **0.59** | **0.86** | **0.71** | **0.59** | **0.85** |
|  |  |  |  |  |  |  |  |  |  |  |  |  |
| **Gender** (Women compared to Men) | **1.17** | **1.03** | **1.32** | **1.11** | **0.98** | **1.25** | **1.17** | **1.04** | **1.33** | **1.21** | **1.07** | **1.38** |
|  |  |  |  |  |  |  |  |  |  |  |  |  |
| **DEPRESSIVE STATE^d^** |  |  |  |  |  |  |  |  |  |  |  |  |
|  | **1.98** | **1.70** | **2.30** | **1.97** | **1.69** | **2.29** | **2.00** | **1.71** | **2.31** | **1.92** | **1.65** | **2.24** |
| **POOR SELF-REPORTED HEALTH^e^** |  |  |  |  |  |  |  |  |  |  |  |  |
|  | **1.50** | **1.26** | **1.79** | **1.47** | **1.23** | **1.76** | **1.49** | **1.25** | **1.78** | **1.46** | **1.22** | **1.75** |
| OR: Odds ratios; 95%CI: Confidence interval at 95%; ^a^ Categories are defined from Alcohol Use Disorders Identification scores as follows: Mild (0-7), Dangerous (8-15), Problematic (16-19) and Dependence (20-40), with Mild category as reference; ^b^ Categories of current smokers are defined as follows: Light (1 to 9 cigarettes per day), Moderate (10 to 19) and Heavy (>19) consumers, with never smokers as reference category; ^c^ Reference category is never use;  ^d^ Depressive state was defined as a total score ≥19 at the Center for Epidemiologic Studies Depression (CESD);  ^e^ Self-reported health was used as a binary variable from an 8-points Likert scale. Significant associations are presented in bold (i.e. p<0.05). | | | | | | | | | | | | |

**S1 Table. Associations between alcohol, tobacco and cannabis use and job loss at one-year among 18,879 participants from the CONSTANCES cohort, adjusting for age, gender, self-reported health and depressive symptoms.**
