# Supplementary material for: Alcohol, tobacco and cannabis use are associated with job loss at follow-up: Findings from the CONSTANCES cohort
Source: PLoS One. 2019 Sep 9;14(9):e0222361. doi: 10.1371/journal.pone.0222361 (PMC6733456; doi:10.1371/journal.pone.0222361)
Supplement: S2 Table — (DOCX) [file pone.0222361.s003.docx]

**S2 Table. Associations between tobacco use and job loss at one-year among 18,879 participants from the CONSTANCES cohort, adjusting for age, gender and self-reported health while stratifying for depressive state.**

| **Stratification for depressive state** | **Not depressed** | | | | **Depressed** | | | |
| --- | --- | --- | --- | --- | --- | --- | --- | --- |
|  | **OR** | **95%CI** | | **p value** | **OR** | **95%CI** | | **p value** |
| Never smoker | Ref. | . | . | . | Ref. | . | . | . |
| Former smoker | **1.30** | **1.10** | **1.53** | **0.002** | **1.11** | **0.81** | **1.53** | **0.51** |
| Light smoker | **1.43** | **1.15** | **1.79** | **0.002** | **1.86** | **1.28** | **2.70** | **<0.001** |
| Moderate smoker | **1.69** | **1.30** | **2.19** | **<0.001** | **1.66** | **1.11** | **2.49** | **0.014** |
| Heavy smoker | **2.52** | **1.71** | **3.73** | **<0.001** | 0.74 | 0.34 | 1.58 | 0.433 |
| OR: Odds ratios; 95%CI: Confidence interval at 95%; Categories of current smokers are defined as follows: Light (1 to 9 cigarettes per day), Moderate (10 to 19) and Heavy (>19) consumers, with never smokers as reference category. Adjustments variables were as follows: gender, age in three categories (<30; ≥30 and <50 and ≥50), self-reported health was used as a binary variable from an 8-points Likert scale. Depressive state defined as a total score ≥19 at the Center for Epidemiologic Studies Depression (CESD). Significant associations are presented in bold (i.e. p<0.05). | | | | | | | | |
