# Supplementary material for: Alcohol, tobacco and cannabis use are associated with job loss at follow-up: Findings from the CONSTANCES cohort
Source: PLoS One. 2019 Sep 9;14(9):e0222361. doi: 10.1371/journal.pone.0222361 (PMC6733456; doi:10.1371/journal.pone.0222361)
Supplement: S3 Table — (DOCX) [file pone.0222361.s004.docx]

**S3 Table. Associations between cannabis use and job loss at one-year among 18,879 participants from the CONSTANCES cohort, adjusting for gender, depressive state and self-reported health while stratifying for age.**

| **Stratification for age** | **<30** | | | | **≥30 and <50** | | | | **≥50** | | | |
| --- | --- | --- | --- | --- | --- | --- | --- | --- | --- | --- | --- | --- |
|  | **OR** | **95%CI** | | **p value** | **OR** | **95%CI** | | **p value** | **OR** | **95%CI** | | **p value** |
| Never use | Ref. | . | . | . | Ref. | . | . | . | Ref. | . | . | . |
| Consumption more than 12 months ago | **2.25** | **1.59** | **3.19** | **<0.001** | **1.29** | **1.07** | **1.57** | **0.009** | **1.39** | **1.11** | **1.74** | **0.004** |
| Less than once a month | **1.96** | **1.21** | **3.19** | **0.006** | **2.04** | **1.41** | **2.96** | **<0.001** | 2.02 | 0.95 | 4.29 | 0.067 |
| Once a month or more | **2.99** | **1.92** | **4.66** | **<0.001** | **2.52** | **1.78** | **3.55** | **<0.001** | **5.31** | **2.72** | **10.38** | **<0.001** |
| OR: Odds ratios; 95%CI: Confidence interval at 95%; Adjustments variables were as follows: gender, self-reported health used as a binary variable from an 8-points Likert scale, and depressive state defined as a total score ≥19 at the Center for Epidemiologic Studies Depression (CESD). Significant associations are presented in bold (i.e. p<0.05). | | | | | | | | | | | | |
