# Supplementary material for: Alcohol, tobacco and cannabis use are associated with job loss at follow-up: Findings from the CONSTANCES cohort
Source: PLoS One. 2019 Sep 9;14(9):e0222361. doi: 10.1371/journal.pone.0222361 (PMC6733456; doi:10.1371/journal.pone.0222361)
Supplement: S4 Table — (DOCX) [file pone.0222361.s005.docx]

**S4 Table. Associations between alcohol, tobacco and cannabis use and job loss at one-year among 18,879 participants from the CONSTANCES cohort, adjusting for age, gender, self-reported health and depressive symptoms, with all substances simultaneously entered and while aggregating “dangerous” and “problematic or dependence” categories regarding alcohol use, as well as the “moderate” and “heavy” categories regarding tobacco use.**

|  | **OR** | **95%CI** | | **p value** | **linear trend** | | | |
| --- | --- | --- | --- | --- | --- | --- | --- | --- |
|  |  |  |  |  | **OR** | **95%CI** | | **p value** |
|  |  |  |  |  |  |  |  |  |
| **Alcohol use^a^** |  |  |  |  |  |  |  |  |
| Dangerous, Problematic or Dependence | **1.25** | **1.05** | **1.48** | **0.011** |  |  |  |  |
|  |  |  |  |  |  |  |  |  |
| **Tobacco use^b^** |  |  |  |  |  |  |  |  |
| Former smoker | 1.08 | 0.93 | 1.26 | 0.324 | **1.09** | **1.02** | **1.17** | **0.009** |
| Light smoker | 1.17 | 0.95 | 1.44 | 0.132 |  |  |  |  |
| Moderate or Heavy smoker | **1.31** | **1.06** | **1.61** | **0.014** |  |  |  |  |
|  |  |  |  |  |  |  |  |  |
| **Cannabis use^c^** |  |  |  |  |  |  |  |  |
| Consumption more than 12 months ago | **1.35** | **1.17** | **1.56** | **<0.001** | **1.30** | **1.98** | **1.41** | **<0.001** |
| Less than once a month | **1.62** | **1.23** | **2.15** | **0.001** |  |  |  |  |
| Once a month or more | **2.21** | **1.69** | **2.88** | **<0.001** |  |  |  |  |
| OR: Odds ratios; 95%CI: Confidence interval at 95%; ^a^ Categories are defined from Alcohol Use Disorders Identification scores as follows: Mild (0-7), Dangerous (8-15), Problematic (16-19) and Dependence (20-40), with Mild category as reference; ^b^ Categories of current smokers are defined as follows: Light (1 to 9 cigarettes per day), Moderate (10 to 19) and Heavy (>19) consumers, with never smokers as reference category; ^c^ Reference category is never use. Adjustments variables were as follows: gender, age in three categories (<30; ≥30 and <50 and ≥50), self-reported health was used as a binary variable from an 8-points Likert scale, and depressive state defined as a total score ≥19 at the Center for Epidemiologic Studies Depression (CESD). Significant associations are presented in bold (i.e. p<0.05). | | | | | | | | |
