# Supplementary material for: Alcohol, tobacco and cannabis use are associated with job loss at follow-up: Findings from the CONSTANCES cohort
Source: PLoS One. 2019 Sep 9;14(9):e0222361. doi: 10.1371/journal.pone.0222361 (PMC6733456; doi:10.1371/journal.pone.0222361)
Supplement: S5 Table — (DOCX) [file pone.0222361.s006.docx]

| **Substance** | **Alcohol** | | | **Tobacco** | | | **Cannabis** | | |
| --- | --- | --- | --- | --- | --- | --- | --- | --- | --- |
|  | **OR** | **95%CI** | | **OR** | **95%CI** | | **OR** | **95%CI** | |
| **SUBSTANCE USE** |  |  |  |  |  |  |  |  |  |
| **Alcohol use^a^** | **1.52** | **1.29** | **1.78** |  |  |  |  |  |  |
| **Tobacco use^b^** |  |  |  | **1.47** | **1.28** | **1.69** |  |  |  |
| **Cannabis use^c^** |  |  |  |  |  |  | **1.86** | **1.55** | **2.23** |
|  |  |  |  |  |  |  |  |  |  |
| **SOCIODEMOGRAPHIC FACTORS** |  |  |  |  |  |  |  |  |  |
| **Age** (in years; reference category: Less than 30) |  |  |  |  |  |  |  |  |  |
| Between 30 and 50 | **0.48** | **0.41** | **0.56** | **0.47** | **0.40** | **0.55** | **0.51** | **0.43** | **0.60** |
| More than 50 | **0.60** | **0.51** | **0.72** | **0.60** | **0.50** | **0.71** | **0.67** | **0.56** | **0.80** |
|  |  |  |  |  |  |  |  |  |  |
| **Gender** (Women compared to Men) | **1.16** | **1.02** | **1.32** | 1.09 | 0.96 | 1.23 | 1.13 | 0.99 | 1.28 |
|  |  |  |  |  |  |  |  |  |  |
| **DEPRESSIVE STATE^d^** |  |  |  |  |  |  |  |  |  |
|  | **2.00** | **1.71** | **2.32** | **1.99** | **1.71** | **2.31** | **2.02** | **1.73** | **2.35** |
| **POOR SELF-REPORTED HEALTH^e^** |  |  |  |  |  |  |  |  |  |
|  | **1.51** | **1.26** | **1.80** | **1.50** | **1.26** | **1.80** | **1.52** | **1.27** | **1.81** |
| OR: Odd ratio; 95%CI: Confidence interval at 95%; ISCED: 2011 International Standard Classification of Education; ^a^ Categories are defined from Alcohol Use Disorders Identification scores as follows: Mild (0-7), Dangerous, Problematic and Dependence (8-40), with Mild category as reference; ^b^ Non-smokers is defined as reference category compared to former and current smokers; ^c^ Never used is defined as reference category compared to having already used; ^d^ Depressive state was defined as a total score ≥19 at the Center for Epidemiologic Studies Depression (CESD); ^e^ Self-reported health was used as a binary variable from an 8-points Likert scale.. Significant associations are presented in bold (i.e. p<0.05). | | | | | | | | | |

**S5 Table. Associations between alcohol, tobacco and cannabis use and job loss at one-year among 18,879 participants from the CONSTANCES cohort, adjusting for age, gender, self-reported health and depressive symptoms while using substances as binary variables.**
