# Supplementary material for: Alcohol, tobacco and cannabis use are associated with job loss at follow-up: Findings from the CONSTANCES cohort
Source: PLoS One. 2019 Sep 9;14(9):e0222361. doi: 10.1371/journal.pone.0222361 (PMC6733456; doi:10.1371/journal.pone.0222361)
Supplement: S6 Table — (DOCX) [file pone.0222361.s007.docx]

| **Substances** | **Alcohol** | | | **Tobacco** | | | **Cannabis** | | |
| --- | --- | --- | --- | --- | --- | --- | --- | --- | --- |
|  | **OR** | **95%CI** | | **OR** | **95%CI** | | **OR** | **95%CI** | |
| **SUBSTANCE USE** |  |  |  |  |  |  |  |  |  |
| **Alcohol use^a^** |  |  |  |  |  |  |  |  |  |
| Dangerous | **1.50** | **1.27** | **1.76** |  |  |  |  |  |  |
| Problematic or Dependence | **2.13** | **1.49** | **3.05** |  |  |  |  |  |  |
|  |  |  |  |  |  |  |  |  |  |
| **Tobacco use^b^** |  |  |  |  |  |  |  |  |  |
| Former smoker |  |  |  | **1.26** | **1.10** | **1.44** |  |  |  |
| Light smoker |  |  |  | **1.52** | **1.27** | **1.80** |  |  |  |
| Moderate smoker |  |  |  | **1.84** | **1.50** | **2.26** |  |  |  |
| Heavy smoker |  |  |  | **1.73** | **1.20** | **2.50** |  |  |  |
|  |  |  |  |  |  |  |  |  |  |
| **Cannabis use^c^** |  |  |  |  |  |  |  |  |  |
| Consumption more than 12 months ago |  |  |  |  |  |  | **1.38** | **1.22** | **1.56** |
| Less than once a month |  |  |  |  |  |  | **1.76** | **1.36** | **2.28** |
| Once a month or more |  |  |  |  |  |  | **2.64** | **2.09** | **3.34** |
|  |  |  |  |  |  |  |  |  |  |
| **SOCIODEMOGRAPHIC FACTORS** |  |  |  |  |  |  |  |  |  |
| **Age** (in years) |  |  |  |  |  |  |  |  |  |
| Between 30 and 50 | **0.48** | **0.42** | **0.56** | **0.46** | **0.39** | **0.53** | **0.52** | **0.44** | **0.61** |
| More than 50 | **0.65** | **0.55** | **0.77** | **0.61** | **0.52** | **0.72** | **0.76** | **0.64** | **0.90** |
|  |  |  |  |  |  |  |  |  |  |
| **Gender** (Women compared to Men) | **1.17** | **1.03** | **1.31** | 1.10 | 0.98 | 1.23 | **1.16** | **1.03** | **1.30** |
|  |  |  |  |  |  |  |  |  |  |
| **DEPRESSIVE STATE^d^** |  |  |  |  |  |  |  |  |  |
|  | **1.99** | **1.72** | **2.29** | **1.98** | **1.71** | **2.29** | **2.00** | **1.73** | **2.31** |
| **POOR SELF-REPORTED HEALTH^e^** |  |  |  |  |  |  |  |  |  |
|  | **1.56** | **1.32** | **1.85** | **1.53** | **1.29** | **1.81** | **1.56** | **1.32** | **1.84** |
| OR: Odds ratios; 95%CI: Confidence interval at 95%; ^a^ Categories are defined from Alcohol Use Disorders Identification scores as follows: Mild (0-7), Dangerous (8-15), Problematic (16-19) and Dependence (20-40), with Mild category as reference; ^b^ Categories of current smokers are defined as follows: Light (1 to 9 cigarettes per day), Moderate (10 to 19) and Heavy (>19) consumers, with never smokers as reference category; ^c^ Reference category is never use;  ^d^ Depressive state was defined as a total score ≥19 at the Center for Epidemiologic Studies Depression (CESD);  ^e^ Self-reported health was used as a binary variable from an 8-points Likert scale. Significant associations are presented in bold (i.e. p<0.05). | | | | | | | | | |

**S6 Table. Associations between alcohol, tobacco and cannabis use and job loss over a three-year follow-up among 18,879 participants from the CONSTANCES cohort, adjusting for age, gender, poor self-reported health and depressive state.**
