# Supplementary material for: Alcohol, tobacco and cannabis use are associated with job loss at follow-up: Findings from the CONSTANCES cohort
Source: PLoS One. 2019 Sep 9;14(9):e0222361. doi: 10.1371/journal.pone.0222361 (PMC6733456; doi:10.1371/journal.pone.0222361)
Supplement: S7 Table — (DOCX) [file pone.0222361.s008.docx]

**S7 Table. Associations between alcohol, tobacco and cannabis use and job loss at one-year among 18,225 participants from the CONSTANCES cohort, adjusting for age, gender, education level, occupational grade, household income, effort-reward imbalance ratio, type of contract, type of work time, past time unemployed in the past three years, depressive symptoms and self-reported health.**

| **Substances** | **Alcohol** | | | **Tobacco** | | | **Cannabis** | | |
| --- | --- | --- | --- | --- | --- | --- | --- | --- | --- |
|  | **OR** | **95%CI** | | **OR** | **95%CI** | | **OR** | **95%CI** | |
| **SUBSTANCE USE** |  |  |  |  |  |  |  |  |  |
| **Alcohol use^a^** |  |  |  |  |  |  |  |  |  |
| Dangerous | **1.37** | **1.15** | **1.64** |  |  |  |  |  |  |
| Problematic or Dependence | **1.61** | **1.11** | **2.34** |  |  |  |  |  |  |
|  |  |  |  |  |  |  |  |  |  |
| **Tobacco use^b^** |  |  |  |  |  |  |  |  |  |
| Former smoker |  |  |  | **1.22** | **1.05** | **1.42** |  |  |  |
| Light smoker |  |  |  | **1.38** | **1.14** | **1.68** |  |  |  |
| Moderate smoker |  |  |  | **1.42** | **1.12** | **1.78** |  |  |  |
| Heavy smoker |  |  |  | **1.44** | **1.00** | **2.09** |  |  |  |
|  |  |  |  |  |  |  |  |  |  |
| **Cannabis use^c^** |  |  |  |  |  |  |  |  |  |
| Consumption more than 12 months ago |  |  |  |  |  |  | **1.34** | **1.17** | **1.54** |
| Less than once a month |  |  |  |  |  |  | **1.51** | **1.14** | **2.00** |
| Once a month or more |  |  |  |  |  |  | **2.11** | **1.63** | **2.72** |
|  |  |  |  |  |  |  |  |  |  |
| **SOCIODEMOGRAPHIC FACTORS** |  |  |  |  |  |  |  |  |  |
| **Age** (in years; reference category: Less than 30) |  |  |  |  |  |  |  |  |  |
| Between 30 and 50 | **0.73** | **0.61** | **0.88** | **0.70** | **0.58** | **0.83** | **0.75** | **0.63** | **0.90** |
| More than 50 | 1.03 | 0.85 | 1.25 | 0.97 | 0.80 | 1.18 | 1.13 | 0.92 | 1.38 |
|  |  |  |  |  |  |  |  |  |  |
| **Gender** (Women compared to Men) | 0.90 | 0.78 | 1.03 | **0.86** | **0.75** | **0.98** | **0.90** | **0.79** | **1.03** |
|  |  |  |  |  |  |  |  |  |  |
| **Education ISCED classification^d^** (reference category: Levels 0 to 4) |  |  |  |  |  |  |  |  |  |
| Levels 5 and 6 | 0.98 | 0.83 | 1.15 | 0.99 | 0.84 | 1.16 | **0.97** | **0.82** | **1.13** |
| Levels 7 and 8 | 0.99 | 0.81 | 1.22 | 1.02 | 0.83 | 1.25 | **0.97** | **0.79** | **1.20** |
|  |  |  |  |  |  |  |  |  |  |
| **Occupational grade** (reference category: Blue-collar worker, craftsman or employee) |  |  |  |  |  |  |  |  |  |
| Intermediate worker | **0.72** | **0.61** | **0.86** | **0.73** | **0.61** | **0.86** | **0.72** | **0.60** | **0.85** |
| Executive | **0.82** | **0.68** | **0.99** | 0.83 | 0.69 | 1.01 | **0.82** | **0.68** | **0.99** |
|  |  |  |  |  |  |  |  |  |  |
| **Household income** (More than 2800 euros per month compared to less than 2800) | **0.66** | **0.57** | **0.76** | **0.66** | **0.57** | **0.76** | **0.68** | **0.59** | **0.78** |
|  |  |  |  |  |  |  |  |  |  |
| **OCCUPATIONAL FACTORS** |  |  |  |  |  |  |  |  |  |
| **Effort-reward imbalance ratio^e^** (Third tertile compared to first and second tertiles) | **1.41** | **1.24** | **1.62** | **1.41** | **1.23** | **1.62** | **1.42** | **1.24** | **1.62** |
|  |  |  |  |  |  |  |  |  |  |
| **Type of contract** (Other type of contract compared to open-ended contract) | **1.82** | **1.57** | **2.10** | **1.82** | **1.57** | **2.11** | **1.77** | **1.52** | **2.05** |
|  |  |  |  |  |  |  |  |  |  |
| **Type of work time** (Full-time compared to part-time) | **2.03** | **1.75** | **2.36** | **2.04** | **1.76** | **2.38** | **2.04** | **1.75** | **2.37** |
|  |  |  |  |  |  |  |  |  |  |
| **Past time unemployed in the last three years** (Yes compared to no) | **3.73** | **3.20** | **4.34** | **3.69** | **3.17** | **4.30** | **3.67** | **3.14** | **4.27** |
|  |  |  |  |  |  |  |  |  |  |
| **DEPRESSIVE STATE^e^** |  |  |  |  |  |  |  |  |  |
|  | **1.65** | **1.41** | **1.94** | **1.66** | **1.41** | **1.94** | **1.66** | **1.42** | **1.95** |
| **POOR SELF-REPORTED HEALTH^f^** |  |  |  |  |  |  |  |  |  |
|  | **1.37** | **1.14** | **1.64** | **1.36** | **1.13** | **1.64** | **1.36** | **1.13** | **1.63** |
| OR: Odds ratios; 95%CI: Confidence interval at 95%; ISCED: 2011 International Standard Classification of Education; ^a^ Categories are defined from Alcohol Use Disorders Identification scores as follows: Mild (0-7), Dangerous (8-15), Problematic (16-19) and Dependence (20-40), with Mild category as reference; ^b^ Categories of current smokers are defined as follows: Light (1 to 9 cigarettes per day), Moderate (10 to 19) and Heavy (>19) consumers, with never smokers as reference category; ^c^ Reference category is never use;  ^d^ Computed from 7 items regarding rewards and from 3 items regarding efforts and with all the items assessed on a 4-points Likert scale; ^e^ Depressive state was defined as a total score ≥19 at the Center for Epidemiologic Studies Depression (CESD);  ^f^ Self-reported health was used as a binary variable from an 8-points Likert scale. Significant associations are presented in bold (i.e. p<0.05). | | | | | | | | | |
